# Real-world evidence on long COVID-19 in Greece: A multicenter, cross-sectional study (LONCOV2)

**DOI:** 10.1016/j.ijregi.2025.100761

**Published:** 2025-09-13

**Authors:** Garyphallia Poulakou, Vasileios Michailidis, Athina Gogali, Stylianos Boutlas, Melina Kavousanaki, Paschalina Giouleka, Alexandros Stefanidis, Panagiota Styliara, Paschalis Steiropoulos, Argyris Tzouvelekis

**Affiliations:** 1 3rd Department of Internal Medicine and Laboratory, School of Medicine, National and Kapodistrian University of Athens, Athens General Hospital for Chest Diseases “Sotiria”, Athens, Greece; 2 Pulmonology Department, St. Luke’s Hospital, Thessaloniki, Greece; 3 Respiratory Medicine Department, School of Medicine, University of Ioannina, University Hospital of Ioannina, Ioannina, Greece; 4 Department of Respiratory Medicine, Faculty of Medicine, University of Thessaly, General University Hospital of Larissa, Larissa, Greece; 5 1st Department of Internal Medicine, General Hospital of Heraklion "Venizeleio-Pananeio", Heraklion, Crete, Greece; 6 Pulmonary Department, Bioclinic Thessaloniki, Thessaloniki, Greece; 7 1st Cardiology Department, General Hospital of Nikaia Piraeus “Agios Panteleimon”, Piraeus, Greece; 8 Menarini Hellas S.A., Athens, Greece; 9 Department of Pulmonology, Medical School, Democritus University of Thrace, University General Hospital of Alexandroupolis, Alexandroupolis, Greece; 10 Department of Respiratory Medicine, University Hospital of Patras, Patras, Greece

**Keywords:** SARS-CoV-2, Long COVID, Long-term COVID-19 complications, Multisystem involvement, Multidisciplinary management

## Abstract

**Objectives:**

This study aimed to provide real-world data on the clinical presentation and management of long COVID in Greece.

**Methods:**

This non-interventional, nationwide, multicenter, cross-sectional study included adults with a history of symptomatic SARS-CoV-2 infection who presented with suspected long-term COVID-19 manifestations ≥4 weeks after acute infection.

**Results:**

Among 1011 patients (mean ± SD age: 55.95 ± 15.74 years; 56.18% female; 5.04% hospitalized) enrolled between December 2022 and May 2023, the most affected Medical Dictionary for Regulatory Activities (MedDRA) System Organ Class (SOC) was General disorders and administration site conditions (75.67%), with fatigue/malaise as the predominant symptom (69.93%). This was followed by Respiratory, thoracic, and mediastinal disorders (60.34%), with cough (50.64%) and dyspnea (24.83%) as leading symptoms. Social circumstances, specifically impairments in daily living activities (44.21%), Nervous system disorders (40.26%), mainly headache (23.24%), and Musculoskeletal and connective tissue disorders (27.70%), mainly myalgia (24.63%), were also prominent. The majority (74.38%) had been vaccinated prior to infection, with vaccination shown to be protective against nervous and musculoskeletal symptoms. Females were more prone to systemic, psychiatric, nervous, and musculoskeletal symptoms and impairments in daily activities, but less prone to respiratory symptoms.

**Conclusions:**

Multisystem long-term COVID-19 complications were observed, underscoring the importance of multidisciplinary management of this complex, multifaceted condition.

## Highlights

• Real-world data on long COVID were collected from over 1000 adults in Greece.
• Fatigue/malaise (69.9%) was the most common long-term COVID-19 manifestation.
• Respiratory, nervous, and musculoskeletal symptoms, and daily living issues were common.
• Most patients (74.4%) had been vaccinated prior to infection.
• Vaccination was protective against nervous and musculoskeletal symptoms.
• Multisystem involvement supports the need for multidisciplinary long COVID care.

## Introduction

SARS-CoV-2, responsible for COVID-19, has spread globally as a pandemic, with over 770 million confirmed cases and more than seven million deaths reported worldwide as of November 2024 [1]. The clinical presentation of COVID-19 varies widely, ranging from asymptomatic cases to severe, life-threatening illness [2].

While most individuals recover fully within two to four weeks, a growing body of evidence indicates that acute SARS-CoV-2 infection can result in long-term sequelae involving both the pulmonary system and a broad array of extrapulmonary organ systems, collectively termed post-COVID conditions (also known as long COVID) [3,4]. Recent meta-analyses estimate that the global prevalence of long COVID among individuals with a history of COVID-19 is as high as 45%, though substantial variability exists across studies [[5], [6], [7]].

Defining the clinical presentation of long COVID has proven challenging due to its diverse and fluctuating manifestations. Consequently, varying definitions, differing mainly in the timing of post-infection symptom onset, have been proposed by major health organizations, including the United States (US) Centers for Disease Control and Prevention (CDC), the World Health Organization (WHO) and the United Kingdom (UK) National Institute for Health and Clinical Excellence (NICE) [[8], [9], [10]]. The most common clinical manifestations of long COVID encompass systemic symptoms such as fatigue and malaise; neuropsychiatric symptoms, including brain fog, sleep disorders, memory impairment, anxiety, and depression; respiratory symptoms such as dyspnea and cough; cardiac symptoms, including chest pain and palpitations; and musculoskeletal symptoms, primarily myalgia and arthralgia. These symptoms can range from mild to severe, often require comprehensive care, and in some cases may even result in disability [5,[11], [12], [13]].

Although the pathophysiological mechanisms underlying long COVID remain poorly understood [14,15], certain risk factors have been identified*.* These include female gender, older age, higher body mass index (BMI), smoking, and pre-existing conditions such as anxiety, depression, asthma, chronic obstructive pulmonary disease (COPD), diabetes, ischemic heart disease, and immunosuppression. A history of hospitalization or intensive care unit (ICU) admission has also been associated with an increased risk of developing long COVID [16,17]*.* Conversely, vaccination has demonstrated a protective effect [16,18,19].

Five years since the onset of the COVID-19 pandemic, long COVID remains a major public health concern and continues to hold significant scientific interest, underscoring the urgent need to deepen our understanding of its underlying mechanisms, clinical presentation, and management. The persistent challenge of diagnosing this condition, characterized by its diverse and often multisystemic manifestations, further highlights the importance of addressing these gaps to guide comprehensive care strategies and improve patient outcomes. Within this context, the present study aimed to gather real-world data on the profile of this often “vague” health condition and to examine how it is perceived and managed in routine clinical practice in Greece.

## Methods

### Study design

**LONCOV2** (“**L**ong-term outcomes of C**O**VID-19 in Greece. A **N**on-interventional, multicenter, **C**ross-sectional epidemiological study to provide real-w**O**rld evidence on long-sequelae of SARS-Co**V**-**2** infection”) was a non-interventional, nationwide, multicenter, cross-sectional study involving Greek adults with a history of laboratory-confirmed symptomatic SARS-CoV-2 infection, who had recovered from the acute phase and presented with suspected long-term COVID-19 manifestations. The study was conducted between December 16, 2022, and May 4, 2023, by 120 investigators across Greece, comprising 108 private-sector primary healthcare physicians and 12 hospital-based physicians (internists, general practitioners, cardiologists, and pulmonologists), based either in the Athens Metropolitan area (40.6%) or in other prefectures of the country (59.4%).

### Study population

Eligible participants were adults (≥18 years, male or female) with an established diagnosis of acute symptomatic SARS-CoV-2 infection (confirmed by antigen test and/or reverse transcriptase-polymerase chain reaction [RT-PCR]) within the past six months, who had recovered from the acute phase of the infection and presented with suspected long-term COVID-19 manifestations, These were defined as new, persistent, worsening, or recurrent symptoms or signs present at least four weeks after the acute infection onset [10], as determined by their physician during a routine clinical visit unrelated to COVID-19 (a “random” visit) or during an initial or follow-up clinical assessment of their post-acute infection status, initiated either by the physician or at the patient’s request. Exclusion criteria included a positive SARS-CoV-2 laboratory test (antigen and/or RT-PCR) at the time of the study visit or participation in an interventional study.

### Data collection

The study collected data during a single study visit (enrolment visit), coinciding with a routine clinical visit as defined above. Data were collected through routine clinical and laboratory assessments, medical chart abstraction, and patient self-report using an electronic data capture (EDC) system. No additional diagnostic, monitoring, or therapeutic procedures beyond those of routine clinical practice were performed. Data collected included patient demographics, acute SARS-CoV-2 infection history (e.g., clinical manifestations, severity, vaccination status, hospitalization status, reinfection status), comorbidities, medications, and suspected long-term COVID-19 manifestations (new, persistent, recurrent, or worsening). A predefined list of suspected long-term COVID-19 manifestations, based on prior evidence of post-acute sequelae, was utilized. Information on any recommended follow-up diagnostic and therapeutic plans for suspected long COVID after the study visit was also recorded.

### Study outcomes

The primary outcome of the study was the prevalence of suspected long-term COVID-19 manifestations (symptoms and/or signs), categorized using the Medical Dictionary for Regulatory Activities (MedDRA) System Organ Class (SOC) and Preferred Term (PT) or Lowest Level Term (LLT). Secondary outcomes included potential associations between such manifestations and acute SARS-CoV-2 infection or patient characteristics, as well as recommended diagnostic and/or therapeutic strategies.

### Statistical methods

Statistical analyses were conducted using R version 4.3.1 (R Core Team, Austria). All analyses were based on the full analysis set, comprising all patients who had provided informed consent. Continuous variables are summarized as mean, standard deviation (SD), median, and range, while categorical variables are presented as absolute and relative frequencies with 95% confidence intervals (CIs). For the primary outcome, relative frequencies are presented with Wilson score 95% CIs, using continuity correction. Logistic regression was used to identify associations for the secondary outcomes, with the presence of each long-term COVID-19 manifestation (MedDRA SOC) as the dependent variable (provided the prevalence exceeded 15%), and acute infection characteristics (severity [mild/moderate vs severe/critical], vaccination status [unvaccinated vs vaccinated], hospitalization status [outpatient vs inpatient], patient characteristics (age, gender [male vs female], smoking status [current vs ex-smoker/current vs non-smoker], BMI [normal vs overweight/normal vs obese]), and the presence of comorbidities (COPD, heart failure, hypertension, diabetes mellitus, coronary artery disease, stroke, asthma, autoimmune disease, and cancer) included as fixed factors. The magnitude of associations was expressed as odds ratios (ORs) with 95% CIs and *P*-values. A *P*-value <0.05 was considered statistically significant.

## Results

### Patient demographics and clinical characteristics

A total of 1011 patients were enrolled in this study, with a mean ± SD age of 55.95 ± 15.74 years, and the majority were female (56.18%) (Table 1). The mean BMI was 27.26 ± 4.89 kg/m², with most patients classified as either overweight or obese (65.18%). Over half of the patients were non-smokers (55.19%), while 22.95% were ex-smokers and 21.86% were actively smoking. At the time of the study visit, 51.53% had at least one comorbidity, with hypertension (29.18%) and dyslipidemia (24.63%) being the most common.

**Table 1:** Demographics and comorbidity profile of patients with suspected long COVID, full analysis set.

	*N* = 1011
**Age (years),** mean ± SD	55.95 ± 15.74
**Gender,** n (%)	
Male	443 (43.82)
Female	568 (56.18)
**BMI (kg/m^2^),**^a^ mean ± SD	27.26 ± 4.89
Normal	352 (34.82%)
Overweight	414 (40.95%)
Obese	245 (24.23%)
**Smoking status,** n (%)	
Non-smoker	558 (55.19%)
Ex-smoker	232 (22.95%)
Current smoker	221 (21.86%)
**Comorbidities,** n (%)	
Hypertension	295 (29.18%)
Dyslipidemia	249 (24.63%)
Diabetes mellitus	102 (10.09%)
Asthma	96 (9.50%)
Chronic obstructive pulmonary disease	72 (7.12%)
Coronary artery disease	44 (4.35%)
Heart failure	44 (4.35%)
Autoimmune disease	34 (3.36%)
Cancer	34 (3.36%)
Neuromusculoskeletal disease	14 (1.38%)
Renal dysfunction/impairment	14 (1.38%)
Peripheral artery disease	10 (0.99%)
Stroke	9 (0.89%)
Other	74 (7.32%)

a BMI categories: Normal: BMI <25 kg/m^2^; Overweight: BMI = 25–<30 kg/m^2^; Obese: BMI ≥30 kg/m^2^. BMI, body mass index.

### Acute SARS-CoV-2 infection history

The median time since the onset of acute SARS-CoV-2 infection was 2.7 months (range: 0.9-5.9). For most patients (83.28%), this was their first infection, while 16.72% had a history of at least one prior infection (Table 2). The vast majority (94.96%) were managed on an outpatient basis, indicating mild infection, with only 5.04% requiring hospitalization. A very small proportion (0.49%) were admitted to the ICU, with a median ICU stay of 12 days (range: 7-15).

**Table 2:** Acute SARS-CoV-2 infection history, full analysis set.

	*N* = 1011
**Hospitalization status**, n (%)	
Outpatient	960 (94.96%)
Inpatient	51 (5.04%)
**ICU admission**, n (%)	5 (0.49%)
**Severity,**^a^ n (%)	
Mild/Moderate	993 (98.22%)
Severe/Critical	18 (1.78%)
**First-time SARS-CoV-2 infection**, n (%)	
Yes	842 (83.28%)
No	169 (16.72%)
**Number of previous infections**, n (%)	
1	120 (11.87%)
2	42 (4.15%)
3	4 (0.40%)
More than 3	3 (0.30%)
**Vaccination status**, n (%)	
Unvaccinated	259 (25.62%)
Vaccinated	752 (74.38%)
**Number of vaccine doses**, n (%)	
1 dose	25 (2.47%)
2 doses	115 (11.37%)
3 doses	491 (48.57%)
4 doses	111 (10.98%)
5 doses	10 (0.99%)
**Clinical manifestations**, n (%)	
Fever or chills	792 (78.34%)
Cough	791 (78.24%)
Fatigue	702 (69.44%)
Myalgia (muscle or body aches)	590 (58.36%)
Headache	516 (51.04%)
Oropharyngeal pain (sore throat)	348 (34.42%)
Arthralgia	314 (31.06%)
Nasal congestion/ rhinorrhea (runny nose)	289 (28.59%)
Dyspnea (shortness of breath)	279 (27.60%)
Ageusia/anosmia (loss of taste or smell)	261 (25.82%)
Chest pain	242 (23.94%)
Diarrhea	62 (6.13%)
Nausea or vomiting	47 (4.65%)
Other	23 (2.27%)

ICU, Intensive care unit.

a For this study, severity was defined based on the level of medical care required as follows: mild/moderate (no hospital admission [outpatient care] or non-ICU hospitalization lasting <10 days) and severe/critical (non-ICU hospitalization lasting ≥10 days or requiring ICU admission).

COVID-19 vaccination prior to the acute infection was common, with 74.38% of patients having received at least one dose (Table 2). The median number of doses was 3 (range: 1-5), with 48.57% receiving exactly three doses. The most common clinical manifestations of acute infection, observed in over 50% of patients, were fever or chills (78.34%), cough (78.24%), fatigue (69.44%), myalgia (58.36%), and headache (51.04%).

### Suspected long COVID manifestations

**Figure 1:**
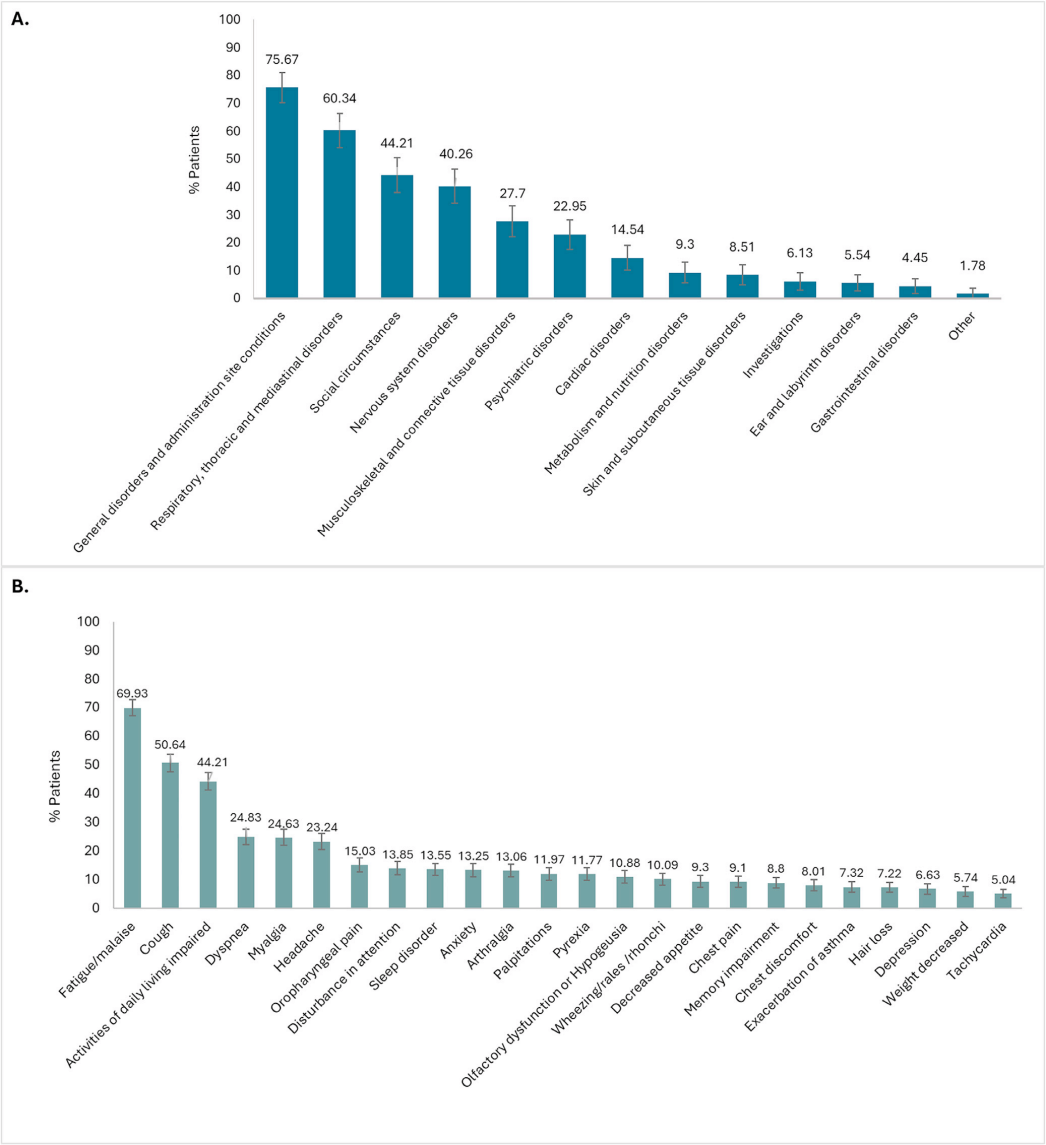
Prevalence of suspected long COVID manifestations (a) by MedDRA system organ class and (b) by MedDRA PT/LLT (≥5%).

LLT, lowest preferred term; MedDRA, Medical Dictionary for Regulatory Activities; PT, preferred term.

The prevalence of specific long-term clinical manifestations suspected to be associated with long COVID, categorized by MedDRA SOC and PT/LLT, is presented in Figure 1 and Table 3. The most commonly affected SOC was General disorders and administration site conditions (75.67%), with fatigue/malaise as the predominant symptom, affecting 69.93% of patients (mild: 49.65%; moderate: 16.82%; severe: 3.46%).

**Table 3:** Suspected long COVID manifestations by MedDRA SOC and PT/LLT, full analysis set.

SOC category		Symptom/sign (PT/LLT)	n	% (95% CI)	New	Persistent	**Relapsed**	**Worsening**
**General disorders and administration site conditions**		Fatigue/Malaise	707	69.93% (66.98–72.73%)	173 (17.11%)	458 (45.30%)	64 (6.33%)	12 (1.19%)
**n**	**% (95% CI)**	Pyrexia	119	11.77% (9.88–13.96%)	48 (4.75%)	34 (3.36%)	30 (2.97%)	7 (0.69%)
765	75.67% (72.88–78.26%)	Chest pain	92	9.10% (7.43–11.08%)	32 (3.17%)	31 (3.07%)	24 (2.37%)	5 (0.49%)
		Chest discomfort	81	8.01% (6.45–9.90%)	30 (2.97%)	33 (3.26%)	12 (1.19%)	6 (0.59%)
		Brain fog	48	4.75% (3.56–6.29%)	16 (1.58%)	26 (2.57%)	3 (0.30%)	3 (0.30%)
**Respiratory, thoracic and mediastinal disorders**		Cough	512	50.64% (47.52–53.77%)	106 (10.48%)	284 (28.09%)	89 (8.80%)	33 (3.26%)
**n**	**% (95% CI)**	Dyspnea	251	24.83% (22.22–27.63%)	70 (6.92%)	130 (12.86%)	38 (3.76%)	13 (1.29%)
610	60.34% (57.24–63.36%)	Oropharyngeal pain	152	15.03% (12.92–17.42%)	38 (3.76%)	67 (6.63%)	36 (3.56%)	11 (1.09%)
		Wheezing/rales/rhonchi	102	10.09% (8.34–12.15%)	29 (2.87%)	44 (4.35%)	21 (2.08%)	8 (0.79%)
		Exacerbation of asthma	74	7.32% (5.83–9.15%)	23 (2.27%)	32 (3.17%)	12 (1.19%)	7 (0.69%)
**Social circumstances**								
**n**	**% (95% CI)**	Activities of daily living impaired	447	44.21% (41.13–47.34%)	118 (11.67%)	272 (26.90%)	38 (3.76%)	19 (1.88%)
447	44.21% (41.13–47.34%)							
**Nervous system disorders**		Headache	235	23.24% (20.70–26.00%)	61 (6.03%)	99 (9.79%)	60 (5.93%)	15 (1.48%)
**n**	**% (95% CI)**	Disturbance in attention	140	13.85% (11.81–16.17%)	51 (5.04%)	74 (7.32%)	7 (0.69%)	8 (0.79%)
407	40.26% (37.23–43.36%)	Olfactory dysfunction /hypogeusia	110	10.88% (9.06–13.00%)	28 (2.77%)	69 (6.82%)	8 (0.79%)	5 (0.49%)
		Memory impairment	89	8.80% (7.16–10.76%)	27 (2.67%)	45 (4.45%)	8 (0.79%)	9 (0.89%)
		Balance disorder	18	1.78% (1.09–2.86%)	6 (0.59%)	8 (0.79%)	3 (0.30%)	1 (0.10%)
		Paresthesia	4	0.40% (0.13–1.08%)	0 (0.00%)	2 (0.20%)	1 (0.10%)	1 (0.10%)
**Musculoskeletal and connective tissue disorders**		Myalgia	249	24.63% (22.03–27.43%)	65 (6.43%)	119 (11.77%)	52 (5.14%)	13 (1.29%)
**n**	**% (95% CI)**	Arthralgia	132	13.06% (11.07–15.33%)	26 (2.57%)	70 (6.92%)	28 (2.77%)	8 (0.79%)
280	27.70% (24.98–30.59%)	Unspecified disorder of joint	6	0.59% (0.24–1.36%)	1 (0.10%)	3 (0.30%)	2 (0.20%)	0 (0.00%)
**Psychiatric disorders**		Sleep disorder	137	13.55% (11.53–15.85%)	49 (4.85%)	65 (6.43%)	18 (1.78%)	5 (0.49%)
**n**	**% (95% CI)**	Anxiety	134	13.25% (11.26–15.54%)	50 (4.95%)	59 (5.84%)	15 (1.48%)	10 (0.99%)
232	22.95% (20.41–25.69%)	Depression	67	6.63% (5.21–8.39%)	22 (2.18%)	23 (2.27%)	12 (1.19%)	10 (0.99%)
**Cardiac disorders**		Palpitations	121	11.97% (10.06–14.17%)	50 (4.95%)	40 (3.96%)	27 (2.67%)	4 (0.40%)
**n**	**% (95% CI)**	Tachycardia	51	5.04% (3.81–6.63%)	20 (1.98%)	23 (2.27%)	6 (0.59%)	2 (0.20%)
147	14.54% (12.37–16.71%)	Postural orthostatic tachycardia syndrome	25	2.47% (1.64–3.68%)	11 (1.09%)	10 (0.99%)	4 (0.40%)	0 (0.00%)
		Bradycardia	6	0.59% (0.24–1.36%)	1 (0.10%)	4 (0.40%)	1 (0.10%)	0 (0.00%)
**Metabolism and nutrition disorders**								
**n**	**% (95% CI)**	Decreased appetite	94	9.30% (7.61–11.30%)	37 (3.66%)	34 (3.36%)	12 (1.19%)	11 (1.09%)
94	9.30% (7.61–11.30%)							
**Skin and subcutaneous tissue disorders**		Hair loss	73	7.22% (5.74–9.04%)	33 (3.26%)	26 (2.57%)	4 (0.40%)	10 (0.99%)
**n**	**% (95% CI)**	Rash	12	1.19% (0.64–2.13%)	6 (0.59%)	4 (0.40%)	1 (0.10%)	1 (0.10%)
86	8.51% (6.90–10.44%)	Nail disorder	6	0.59% (0.24–1.36%)	3 (0.30%)	2 (0.20%)	1 (0.10%)	0 (0.00%)
**Investigations**		Weight decreased	58	5.74% (4.42–7.40%)	27 (2.67%)	19 (1.88%)	5 (0.49%)	7 (0.69%)
**n**	**% (95% CI)**	Cardiac murmur	5	0.49% (0.18–1.22%)	1 (0.10%)	2 (0.20%)	2 (0.20%)	0 (0.00%)
62	6.13% (4.77–7.84%)							
**Ear and labyrinth disorders**		Vertigo	33	3.26% (2.29–4.61%)	14 (1.38%)	11 (1.09%)	5 (0.49%)	3 (0.30%)
**n**	**% (95% CI)**	Hearing loss	22	2.18% (1.40–3.33%)	7 (0.69%)	11 (1.09%)	2 (0.20%)	2 (0.20%)
56	5.54% (4.25–7.18%)	Tinnitus	9	0.89% (0.44–1.75%)	3 (0.30%)	3 (0.30%)	2 (0.20%)	1 (0.10%)
**Gastrointestinal disorders**		Nausea	24	2.37% (1.56–3.57%)	14 (1.38%)	2 (0.20%)	4 (0.40%)	4 (0.40%)
**n**	**% (95% CI)**	Gastrointestinal motility disorder	22	2.18% (1.40–3.33%)	7 (0.69%)	7 (0.69%)	3 (0.30%)	5 (0.49%)
45	4.45% (3.30, 5.96%)	Abdominal pain	16	1.58% (0.94–2.62%)	7 (0.69%)	6 (0.59%)	2 (0.20%)	1 (0.10%)
**Other symptoms/signs**			18	1.78% (1.09–2.86%)	5 (0.49%)	9 (0.89%)	4 (0.40%)	0 (0.00%)

CI, confidence interval; LLT, lowest level term; PT, preferred term; SOC, system organ class.

The second most affected SOC was Respiratory, thoracic, and mediastinal disorders (60.34%), with cough (50.64%) and dyspnea (24.83%) as the leading symptoms. Social circumstances, specifically impairments in activities of daily living, followed, with a prevalence of 44.21%. Nervous system disorders affected 40.26% of patients, with headache (23.24%) and attention disturbance (13.85%) being the most common neurological symptoms.

Musculoskeletal and connective tissue disorders affected 27.70% of patients, with myalgia (24.63%) and arthralgia (13.06%) being the most common symptoms, while Psychiatric disorders were noted in 22.95% of patients, with sleep disturbances (13.55%) and anxiety (13.25%) being the most frequent mental health issues. All other SOCs were present in fewer than 15% of patients.

### Acute SARS-CoV-2 infection, demographics, and comorbidities as predictors of long COVID manifestations

Multivariate logistic regression revealed significant associations between patient demographics, acute infection characteristics, certain comorbidities, and the presence of various suspected long COVID manifestations. Hospitalization during acute SARS-CoV-2 infection (inpatient management) was significantly associated with an increased likelihood of developing symptoms within the SOC General disorders and administration site conditions (OR 4.12, 95% CI: 1.20-14.2, *P* = 0.025) and Social circumstances (OR 4.56, 95% CI: 1.81-11.5, *P* = 0.001). In contrast, COVID-19 vaccination prior to acute infection was a protective factor against Nervous system disorders (OR 0.73, 95% CI: 0.55-0.98, *P* = 0.038) and Musculoskeletal and connective tissue disorders (OR 0.62, 95% CI: 0.46-0.85, *P* = 0.003), with unvaccinated individuals more likely to experience such symptoms.

Gender was also a significant factor, with females being more likely to develop General disorders and administration site conditions (OR 1.40, 95% CI: 1.04-1.91, *P* = 0.028), Social circumstances impairments (OR 1.42, 95% CI 1.08-1.87, *P* = 0.012), Psychiatric disorders (OR 1.60, 95% CI: 1.15-2.21, *P* = 0.005) and Musculoskeletal and connective tissue disorders (OR 1.51, 95% CI: 1.11-2.03, *P* = 0.008), but less likely to experience Respiratory, thoracic and mediastinal disorders (OR 0.73, 95% CI: 0.55-0.96, *P* = 0.026).

Increasing age was associated with a higher risk of General disorders and administration site conditions (OR 0.99, 95% CI: 0.98-1.00, *P* = 0.023) and Respiratory, thoracic and mediastinal disorders (OR 1.01, 95% CI: 1.00-1.02, *P* = 0.030). Non-smokers were found to have a reduced risk of developing Nervous system disorders (OR 0.65, 95% CI: 0.47-0.90, *P* = 0.009) and Musculoskeletal and connective tissue disorders compared to current smokers (OR 0.64, 95% CI: 0.45-0.92, *P* = 0.014).

Regarding comorbidities, patients with COPD and asthma were significantly more likely to experience Respiratory, thoracic, and mediastinal disorders (OR 2.90, 95% CI: 1.46-5.76, *P* = 0.002, and OR 5.28, 95% CI: 2.87-9.71, *P* < 0.001, respectively). Hypertension was associated with increased risks of both General disorders and administration site conditions (OR 1.59, 95% CI: 1.07-2.36, *P* = 0.020) and Musculoskeletal and connective tissue disorders (OR 1.60, 95% CI: 1.11-2.32, *P* = 0.011). Patients with coronary artery disease (OR 3.24, 95% CI: 1.19-8.80, *P* = 0.021) and autoimmune disease (OR 3.78, 95% CI: 1.13-12.7, *P* = 0.031) were at higher risk of developing General disorders and administration site conditions, and those with a history of stroke or cancer had increased odds of experiencing psychiatric disorders (OR 5.11, 95% CI: 1.20-21.7, *P* = 0.027 and OR 2.45, 95% CI: 1.18-5.06, *P* = 0.016, respectively).

### Diagnostic tests for suspected long COVID symptoms

Nearly one quarter of patients (24.73%) had undergone at least one diagnostic test for suspected long COVID prior to the study visit. The most common tests were complete blood count and biochemical tests (17.90% each), chest X-rays (14.24%), and pulmonary function tests (i.e., spirometry; 11.47%). Other tests, such as chest computed tomography (CT) (5.74%), electrocardiogram (8.41%), and echocardiography (5.84%), were less frequently performed.

### Medications

At the time of the study visit, 45.8% of patients were taking at least one medication for suspected long COVID symptoms, with inhalers being the most common (30.27% overall; 22.65% initiated prior to the study visit and 7.62% during the study visit). Other medications, including angiotensin-converting enzyme inhibitors, angiotensin II receptor blockers, beta-blockers, statins, oral antithrombotics, antidiabetics, oral corticosteroids, and antiarrhythmics, were taken by fewer than 3% of patients.

Additionally, 41.94% of patients were taking at least one medication for conditions unrelated to specific long COVID symptoms. The most commonly used were statins (22.06%), angiotensin-converting enzyme inhibitors (11.37%), and beta-blockers (11.28%).

### Diagnostic and therapeutic follow-up of suspected long-term COVID-19 manifestations

**Figure 2:**
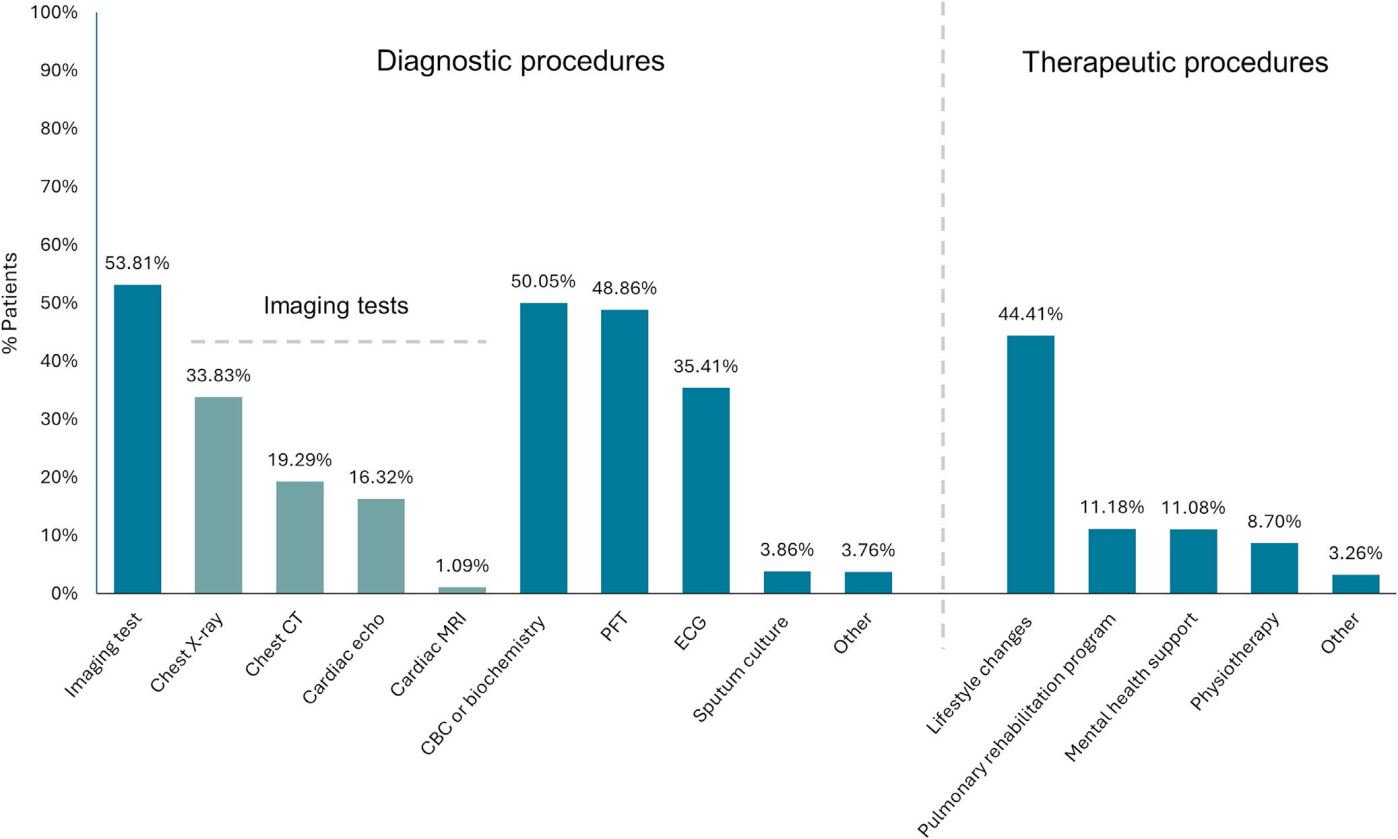
Recommended follow-up diagnostic and therapeutic procedures during the study visit.

CBC, complete blood count; CT, computed tomography; ECG, electrocardiogram; Cardiac echo, cardiac echocardiography; MRI, magnetic resonance imaging; PFT, pulmonary function test.

During the study visit, physicians recommended further diagnostic evaluations for suspected long COVID symptoms, with at least one imaging test in 53.81% of the patients, chest X-ray being the most common (33.83%). Pulmonary function tests (48.86%), complete blood count or biochemical tests (50.05%), and electrocardiogram (35.41%) were also frequently recommended (Figure 2).

Therapeutic recommendations were primarily focused on lifestyle changes (44.41%), whereas pulmonary rehabilitation and mental health support were advised to a smaller subset (11.18% and 11.08%, respectively) (Figure 2). Only 26.90% of patients were referred to other specialists, with cardiologists being the most common referral (14.14%).

## Discussion

This study provides real-world insights into the clinical presentation and management of long COVID in a large cohort of adults with a history of symptomatic SARS-CoV-2 infection, managed in routine clinical practice in Greece. Participants experienced suspected long-term COVID-19 manifestations affecting multiple body organ systems, with General disorders and administration site conditions being the most affected MedDRA SOC (76%), followed by Respiratory, thoracic and mediastinal disorders (60%), Social circumstances (44%), and Nervous system disorders (40%). While similar patterns of multisystem involvement have been documented, few studies have utilized organ systems (e.g., MedDRA SOCs) to describe long COVID manifestations [12,20,21]. These findings align with contemporary evidence recognizing multisystem involvement as a hallmark of long COVID [12,13,15]. By focusing on affected organ systems rather than isolated symptoms, the use of MedDRA SOC terms allows for better capture of the condition’s complexity and multisystemic nature, supporting a multidisciplinary approach to patient care.

Fatigue and malaise emerged as the most prevalent symptoms, affecting approximately 70% of participants, followed by impairments in daily activities (51%) and cough (44%). Dyspnea, myalgia, and headache were less frequent, affecting nearly 25%. These symptoms are consistently documented in the literature [5,6,[11], [12], [13],[20], [21], [22], [23]], and are among those endorsed by both the Centers for Disease Control and Prevention (US) [10] and WHO [9], reinforcing their prominence as core features of long COVID. Focusing on Greece, two smaller studies have been conducted—a single-center observational study using a similar definition for long COVID symptoms and an online survey conducted within a long COVID Greek support group, also reporting fatigue as the leading symptom, with a prevalence closely matching that observed in our cohort [22,23].

Despite the overwhelming volume of research, a clearly defined set of symptoms for accurately identifying long COVID remains elusive, and a universally agreed-upon definition is still lacking [8,24]. This ambiguity introduces considerable variation when translating findings into clinical practice, particularly when determining follow-up timing and standardized diagnostic strategies. In this study, we adopted the CDC’s definition in place at the time [10], defining long COVID symptoms as those extending beyond four weeks and up to six months post-acute infection. Nevertheless, although long COVID symptoms generally decline with time, some may persist for up to three years post-acute infection [25,26], indicating a longer-lasting risk horizon for COVID-19, similar to that observed in previous coronavirus outbreaks [8].

The hospitalization rate during acute infection was low (5%), consistent with evidence that long COVID predominantly affects non-hospitalized patients with mild acute disease, who represent the majority of COVID-19 cases [14]. Therefore, it is of the utmost importance to recognize potentially suspicious long COVID symptoms early and ensure proactive follow-up through multidisciplinary outpatient care. Identifying risk factors for long COVID, such as female gender, older age, pre-existing conditions, and hospitalization during acute disease, is also crucial [16,17]. Our multivariate analyses revealed that females were more likely to experience systemic, psychiatric, and musculoskeletal manifestations, as well as impairments in daily activities, while being less prone to respiratory manifestations. This finding aligns with gender differences reported in large meta-analyses [11,16]. Additionally, hospitalization during acute infection increased the risk of systemic symptoms and impaired daily activities, while smoking was associated with nervous system and musculoskeletal symptoms. Hypertension, present in nearly a third of the cohort, was associated with systemic and musculoskeletal symptoms. Asthma and COPD, though less prevalent, were strongly associated with respiratory symptoms, reflecting the vulnerability of these individuals’ respiratory systems to COVID-19.

The majority of patients (83%) in our study experienced long COVID after their first SARS-CoV-2 infection, aligning with evidence that long COVID is more common following initial infections, except for the Omicron BA variant, where reinfections are more frequently implicated. This corresponds with our study period, dominated by earlier variants such as Alpha and Delta, for which this trend is well-documented [27]. While reinfections are less likely to cause long COVID compared to initial infections, they still carry an added risk for persistent symptoms and contribute to the cumulative burden of the condition [4,28]. Notably, approximately 74% of our cohort had been vaccinated (median three doses) prior to their acute infection. Vaccination has demonstrated an overall protective effect against long COVID [18,19], and our data suggest that it may specifically lower the risk of nervous system and musculoskeletal manifestations, likely by reducing severe disease and accelerating viral clearance [19]. These findings indicate that, although vaccination reduces some risks, it does not fully eliminate the burden of long COVID. Thus, further research is essential to understand its interplay with reinfections and long-term COVID-19 complications to guide effective public health strategies.

During our study, further diagnostic evaluations were recommended for about half of the participants, reflecting the physicians’ balanced approach to addressing suspected long COVID manifestations. Respiratory assessments, including imaging (e.g., X-rays) and spirometry, along with routine laboratory tests, were prioritized, consistent with the high prevalence of respiratory and systemic symptoms. Lifestyle modifications were the most frequently recommended intervention (44%), with notably underutilized strategies such as pulmonary rehabilitation and mental health support (11% each)—both crucial for addressing long COVID’s functional and psychological challenges [29]. Limited referrals to other specialists (26.9%) suggest a reliance on single-specialty management, potentially overlooking the advantages of addressing the multifaceted nature of this condition through a multidisciplinary approach [29].

Overall, with the growing global population of COVID-19 survivors, the impact of long COVID on individuals, healthcare systems, and society is likely substantial [15]. Managing this complex condition is challenging due to the diverse symptoms and multisystem involvement, necessitating a tailored, patient-centered approach. Primary care physicians are central to this effort, playing a pivotal role in screening, diagnosis, and timely referrals to specialized care through well-defined pathways [8,15]. To address long COVID effectively, physicians should remain vigilant for symptoms, especially in high-risk patients, and adopt a comprehensive approach to care that includes mapping current symptoms, assessing COVID-19 history, screening for comorbidities, providing targeted interventions for acute symptoms and underlying conditions, educating patients about potential post-COVID-19 manifestations, ensuring regular follow-up, and encouraging them to seek prompt medical attention if symptoms worsen [8,30].

This study has several limitations inherent to its non-interventional design, including the absence of a control group, selection bias, and information/recall bias. Selection bias at the patient level was mitigated by consecutive enrolment, and at the site level by including both hospital outpatient clinics and private primary care offices. Information/recall bias may arise from patient self-reports, potentially leading to under- or overreporting of clinical manifestations; however, physician assessments were also considered. The cross-sectional design limits the ability to track symptom progression over time and establish causal relationships between risk factors and symptoms. Moreover, as most participants were non-hospitalized during the acute disease, individuals with severe acute disease may be underrepresented, though such cases constitute a minority of the affected population. Limited diagnostic correlations, coupled with potential confounding by comorbidities, suggest that some symptoms may represent exacerbations of pre-existing conditions rather than new manifestations of long COVID. Additionally, the CDC and the National Academies of Sciences, Engineering, and Medicine recently updated the definition of long COVID, now described as a chronic condition arising after SARS-CoV-2 infection and persisting for at least three months [31]. Since our study used the earlier CDC definition with a four-week cutoff, comparability with future studies may be limited. Finally, wide CIs for some associations warrant cautious interpretation and further validation in future studies.

## Conclusion

LONCOV2 is the largest Greek study to date, providing real-world insights into the clinical presentation of long COVID in a geographically diverse adult population with prior symptomatic SARS-CoV-2 infection. Our findings confirm that long COVID involves multisystem complications, with fatigue/malaise as the most prevalent symptom. Addressing these challenges requires multidisciplinary care and tailored support for at-risk populations. Future research should focus on standardizing diagnostic criteria, tracking symptom evolution, and developing integrated care pathways to reduce the societal and healthcare burden of long COVID.

## Funding

The LONCOV2 study was sponsored by Menarini Hellas S.A. The study sponsor was involved in the study conception and design, as well as in the interpretation of the data presented in this publication, but not in data collection or analysis. Menarini Hellas S.A. also funded medical writing support for this publication.

## Ethical approval

The study was conducted in accordance with the Declaration of Helsinki, the International Society for Pharmacoepidemiology’s (ISPE) Guidelines for Good Pharmacoepidemiology Practices (GPPs), and all applicable local laws. Approval was granted by the Ethics Committees (ECs) of coordinating hospitals, and all participants provided written informed consent prior to inclusion. ECs and approval dates:

• EC of University Hospital of Patras (08 Feb 2023)
• EC of General University Hospital of Larissa (01 Dec 2022)
• EC of University Hospital of Ioannina (24 Jan 2023)
• EC of Athens General Hospital for Chest Diseases “Sotiria”: 7th & 9th Pulmonary Departments (05 Dec 2022); 3rd Department of Internal Medicine and Laboratory (23 Dec 2022)
• EC of St. Luke’s Hospital (30 Jan 2023)
• EC of University General Hospital of Alexandroupolis (08 Dec 2022)
• EC of General Hospital of Nikaia Piraeus “Agios Panteleimon” (21 Dec 2022)
• EC of General Hospital of Heraklion “Venizeleio-Pananeio” (20 Dec 2022)
• EC of General Hospital of Piraeus “Tzaneio” (13 Dec 2022)
• EC of Bioclinic Thessaloniki (28 Feb 2023).

## Declaration of competing interest

VM, SB, MK, PG, AS, PS, and AT declare that they have no known competing financial interests or personal relationships that could have appeared to influence the work reported in this paper. VM, SB, and PG are Guest Editors for this journal but were not involved in the editorial review or the decision to publish this article. GP has received grants and/or research support and served as a principal investigator for studies sponsored by Pfizer, MSD, Gilead, Menarini, PharmaMar, Fabentech, Bausch, AstraZeneca, the Hellenic Institute for the Study of Sepsis, University College London, and the University of Minnesota. She has also served on advisory boards for Pfizer, AstraZeneca, Gilead, MSD, Menarini, and SOBI. AG has received honoraria and consultancy fees from Boehringer Ingelheim, Chiesi, ELPEN, GSK, and Menarini. P. Sty. is an employee of Menarini Hellas S.A.
